# Impact of Immune Cell Heterogeneity on HER2+ Breast Cancer Prognosis and Response to Therapy

**DOI:** 10.3390/cancers13246352

**Published:** 2021-12-17

**Authors:** Milena Perrone, Giovanna Talarico, Claudia Chiodoni, Sabina Sangaletti

**Affiliations:** Molecular Immunology Unit, Department of Research, Fondazione IRCCS Istituto Nazionale Tumori, 20133 Milan, Italy; milena.perrone@istitutotumori.mi.it (M.P.); giovanna.talarico@istitutotumori.mi.it (G.T.)

**Keywords:** breast cancer, HER2, tumor microenvironment, immune cells, trastuzumab

## Abstract

**Simple Summary:**

Intra-and inter-tumor heterogeneity characterizes breast cancer disease not only in terms of intrinsic cancer cell features, but also of its surrounding microenvironment that can be characterized by different stromal and immune cell types. Nonetheless, triple-negative breast cancers and HER2+ tumors are considered, among breast cancer molecular subtypes, the most immune infiltrated, and the level of TILs generally indicates a good prognosis. It is now clear that both cancer cell molecular heterogeneity and heterogeneity of the tumor microenvironment contribute to modulating the response to anti-HER2 agents. Uncovering all these levels of complexity will be a critical step in the design of patient-tailored treatments; additionally, recent technological advances in the analysis of tumor tissues, such as ssRNAseq and digital pathology, will be key in this process.

**Abstract:**

Breast cancer is a heterogeneous disease with a high degree of diversity among and within tumors, and in relation to its different tumor microenvironment. Compared to other oncotypes, such as melanoma or lung cancer, breast cancer is considered a “cold” tumor, characterized by low T lymphocyte infiltration and low tumor mutational burden. However, more recent evidence argues against this idea and indicates that, at least for specific molecular breast cancer subtypes, the immune infiltrate may be clinically relevant and heterogeneous, with significant variations in its stromal cell/protein composition across patients and tumor stages. High numbers of tumor-infiltrating T cells are most frequent in HER2-positive and basal-like molecular subtypes and are generally associated with a good prognosis and response to therapies. However, effector immune infiltrates show protective immunity in some cancers but not in others. This could depend on one or more immunosuppressive mechanisms acting alone or in concert. Some of them might include, in addition to immune cells, other tumor microenvironment determinants such as the extracellular matrix composition and stiffness as well as stromal cells, like fibroblasts and adipocytes, that may prevent cytotoxic T cells from infiltrating the tumor microenvironment or may inactivate their antitumor functions. This review will summarize the state of the different immune tumor microenvironment determinants affecting HER2+ breast tumor progression, their response to treatment, and how they are modified by different therapeutic approaches. Potential targets within the immune tumor microenvironment will also be discussed.

## 1. Introduction

Breast cancer (BC) is the most commonly diagnosed malignancy in women worldwide [1]. BC is characterized by high inter-tumor heterogeneity which is based on the different identities of initiating cells undergoing malignant expansion. The result of such biological variability is that every molecular BC subtype is associated with a distinct disease processes characterized by specific patterns of evolution.

In addition to cell-autonomous traits, extrinsic microenvironmental factors can contribute to such tumor heterogeneity. The bidirectional interaction between cancer and the surrounding host cells both supports tumor-specific dynamic changes during malignant cancer progression and promotes cancer cell adaptation to environmental cues [2]. The last is particularly evident in the case of the hypoxic microenvironment that might force the development of metastases [3].

In this scenario, a further level of complexity is added by the existence of an intra-tumor clonal level of heterogeneity in which somatic mutations drive the formation of subclones endowed with different biological properties that can be sustained in their tumorigenicity by specific local factors/cells forming specialized “niches”. In this context, the extracellular matrix might play a relevant role in sustaining pro-tumorigenic signals and favoring the establishment of an immune-suppressive microenvironment [4]. A relevant aspect of intra-tumor heterogeneity is that it evolves in time and space, it changes in response to therapy and development of resistance, and varies among tumor patients.

Therefore, a critical aspect related to the characterization of the tumor microenvironment (TME) is to understand how it evolves and to find out possible common traits that, if conserved upon anti-tumor therapy or during metastatic dissemination, might represent the Achille’s heel of a tumor.

## 2. Cellular Composition of the Tumor Microenvironment

The concept of TME is very old and dates back to the time when the relationship between cancer and inflammation was proposed. Malignant cells are surrounded by a complex microenvironment including fibroblasts, different types of immune cells, blood and lymphatic endothelial cells, and extracellular matrix (ECM) elements, including collagens, proteoglycans, and matricellular proteins [5]. Since the very early works, an incredible number of studies have underlined that the different components of the TME create an intricate network that influences the development, aggressiveness, and cancer progression that endows clinical significance [6]. Immune cells within the TME actively interact with cancer cells but also each other, shaping the multi-directional crosstalk that evolves continuously during tumor progression.

On a cellular level, TME comprises a broad spectrum of subpopulations. Hanahan and Coussens, for simplification, have grouped these into three compartments including angiogenic vascular cells, cancer-associated fibroblastic cells (CAFs), and infiltrating immune cells [7]. In the specific case of BC, another relevant component of the TME is represented by adipocytes. Once considered mere bystander cells, now cancer-associated adipocytes (CAAs) are emerging as key partners favoring BC proliferation, invasion, and metastasis through the secretion of adipokines, such as leptin and adiponectin, and different chemokines and interleukins [8]. The increased leptin secretion by CAAs can promote cell proliferation and tumor angiogenesis. Adiponectin, on the contrary, is decreased in CAAs and plays an anti-tumor role inducing apoptosis and suppressing tumor growth and invasion of BC cells through adenosine monophosphate-activated protein kinase (AMPK) activation and the inhibition of the PI3K/AKT pathway [9].

Angiogenic vascular cells, including pericytes, endothelial cells, and smooth muscle cells, have been extensively reported to contribute to tumor growth by providing nutrients and oxygen [10]. They allow trafficking of growth-promoting factors as well as facilitate cancer cell dissemination and metastasis seeding [11]

CAFs are the most prominent compartment of the TME in BC and are involved in all the steps of carcinogenesis, from initial growth to invasion and metastatic dissemination [12]. Given the physiological role of fibroblasts in wound healing, inflammation, and ECM remodeling, and the fact that tumors are considered “wounds that do not heal”, their presence, mainly in an activated state of the TME, is not surprising. CAFs produce a variety of soluble factors contributing to tumor progression, thereby influencing the majority of the so-called hallmarks of cancer [13,14]. Of note in this context, in a very recent study, Costa and colleagues analyzed CAF heterogeneity in each different molecular BC subtypes through multiparametric flow cytometry and investigated the link of this heterogeneity with the immunosuppressed microenvironmental characteristics [15]. The authors identified four distinct CAF subsets, denominated S1-S4, that were differentially located in the tumor mass and the adjacent healthy tissue. Interestingly, these CAF subsets were differentially enriched in the three BC molecular subtypes they have analyzed: luminal A, HER2-enriched, and TNBC, and were associated with specific immune cell subsets, such as Foxp3+ T regulatory cells (T_regs_) with CAF-S1 [15].

Among infiltrating immune cells, macrophages are one of the most abundant sub-population present at the tumor site and are mainly referred to as tumor-associated macrophages (TAMs). TAMs are generally associated with a poor prognosis due to their ability to promote tumor angiogenesis via the secretion of multiple factors, such as Epidermal Growth Factor (EGF), Vascular-Endothelial Growth Factor (VEGF), Platelet-Derived Growth Factor (PDGF), Tumor Necrosis Factor α (TNFα), C-C Motif Chemokine Ligand 2 (CCL2), and (C-X-C Motif Chemokine Ligand 8) CXCL8, [16], in addition to migration and metastatic spreading through the release of matrix metalloproteinases and chemokines [17], and immunosuppression [18]. Along with macrophages, other infiltrating immune cells populate the TME, such as neutrophils and different T cell subsets, that can have either tumor-promoting or inhibiting activities, depending on the local milieu.

## 3. Tumor Microenvironment Composition in the HER2+ Breast Cancers

**Figure 1:**
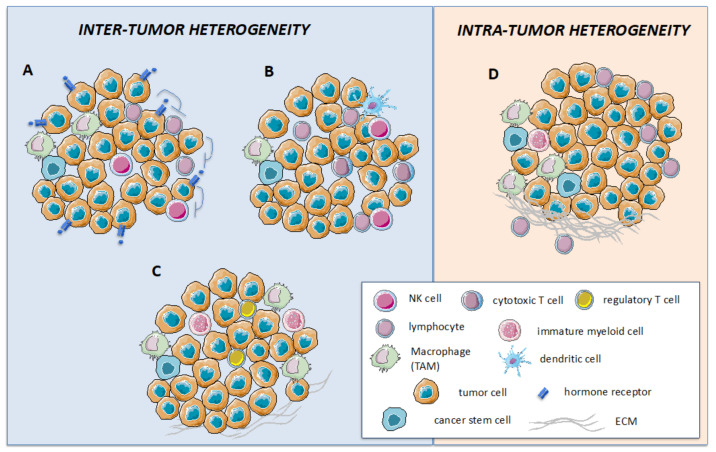
Inter-and intra-tumor heterogeneity dictates the response to HER2-targeted therapies. HER2+ breast cancers are characterized by a high degree of inter- (**A**–**C**) and intra- (**D**) tumor heterogeneity, both contributing to shaping the response to HER2-directed therapies. In the case of inter-tumor heterogeneity, differences may be detected at the level of tumor cells, such as the expression of an estrogen receptor (ER, panel **A**) that, upon HER2 inhibition, functions as an escape/survival pathway. Additionally, intra-tumor heterogeneity may rely on the different immune cell infiltration that may be enriched in effector T and NK cells, leading to a good response to treatment (panel **B**), or in immune-suppressive cells, such as T regulatory cells and tumor associate macrophages (TAMs), leading to poor response (panel **C**). Heterogeneity may also be detected among different regions of the same tumor, with specific areas enriched in the extracellular matrix and TAM/myeloid cells that support the development of micro-niches favoring the selection of resistant neoplastic clones (panel **D**).

The heterogeneity of BC is not limited to the intrinsic features of neoplastic cells, such as morphology, cell of origin, and molecular alterations. Indeed, it is now very clear that TME shows a multifaceted landscape defined by the different cellular as well as non-cellular elements (i.e., ECM) that populate the tumor stroma (Figure 1).

Yet, clinical decision-making for BC treatment is still based on the evaluation of neoplastic cells, assessing hormone receptor (HR) expression and HER2 status, by immunohistochemistry and in situ hybridization (ISH) techniques, respectively [19]. However, we are recently coming to terms that other cells in the TME, in particular those of the immune system, may be relevant not only for BC prognosis but also for predicting response to therapies [20]. Immune cell types infiltrating the tumor stroma not only show inter-tumor heterogeneity but may also be phenotypically/genotypically different in distinct areas of the tumor mass. Despite this great heterogeneity, some general features are characteristics of the different BC molecular subtypes, with HER2+ carcinomas and triple-negative breast cancers (TNBCs) being those with the highest content of immune cell infiltrate. Accordingly, these two BC subsets are considered the more immune-enriched, i.e., hot tumors, in comparison with the luminal carcinomas that are mainly immune-desert, i.e., cold tumors [21]. The concept that tumor-infiltrating lymphocytes (TILs) are a good prognostic indicator for TNBC and HER2+ BCs is generally accepted and they are now being included in clinical diagnostic practice [22,23]. Conversely, the infiltrate mainly enriched by immunoregulatory cells, such as T_regs_ and M2 macrophages, has been associated with poor prognosis [24,25].

Several clinical studies have highlighted the significant role played by immune cells in BCs [23,26,27]. In a key study, Denkert and colleagues analyzed the number of stromal TILs in biopsies from 3771 patients with primary BC treated with neoadjuvant chemotherapy and included in six randomized trials designed by the German Breast Cancer Group [22]. They reported a predictive role of TILs in response to neoadjuvant chemotherapy in all molecular subtypes analyzed, and an association with survival benefit in HER2+ BCs and TNBC. On the other hand, high TILs were a negative prognostic factor for survival in luminal-HER2-negative (HER2-) BC. Of note, the finding in luminal BC may suggest a different biology of TILs in these tumors or, being as luminal tumors are less infiltrated, may just depend on the very scant presence of infiltrating lymphocytes in these tumors.

Despite a number of studies that have investigated the immune infiltration in BC, the composition, localization, and dynamic changes of TILs during and after treatment are still poorly defined. The recent technological advancements in the analysis of the tumor immune contexture will certainly, in the very near future, shed some light on all these issues (see Section 4).

### 3.1. Adaptive Immune Cells

TILs are, by definition, mononuclear immune cells that infiltrate tumor tissue and have been identified in most solid cancers. Specifically, in BC disease, TILs are mainly represented by CD8+ T cells, with a variable, much lower number of CD4+ T cells, B lymphocytes, and rarely, natural killer cells. TILs are generally evaluated by hematoxylin and eosin (H&E) staining on formalin-fixed paraffin-embedded (FFPE) slides by assessment of their typical morphology or by additional immunohistochemistry (IHC) staining for lymphocyte-specific markers. The pathologist usually makes a distinction between stromal TILs, which infiltrate the stroma adjacent to the tumor cells, and intratumoral TILs, which are located inside of the tumor nests in close contact with neoplastic cells. Stromal TILs represent, in general, the vast majority of infiltrating lymphocytes in BC and are more reliable in predicting patient outcome and response to therapy [28]. Importantly, the reliability of using TIL evaluation, as either a prognostic or predictive biomarker, is consistent only if performed with a standardized and reproducible method. Consistent with this recommendation, in 2014, precise guidelines for the evaluation of TILs have been published by an International TILs Working Group [28]. However, TILs evaluation by H&E has some limitations, such as different infiltration and distribution in distinct areas of the tumor, as well as multiple T cell subsets; despite this, its prognostic and predictive value has been widely reported by several independent investigators in the last decade (for a comprehensive review [23]). To mention a few studies on this topic, Sotiriou’s group investigated the correlation between quantity and location of TILs, at diagnosis, with the clinical outcome in more than 2000 node-positive BC samples from the BIG 02-98 adjuvant phase III trial comparing different chemotherapy regimens. While they did not find any prognostic association globally or in the Estrogen Receptor (ER)+/HER2- group, in HER2+ BC they observed a significant interaction between increasing stromal TILs (10% increments) and with chemotherapy benefit based on anthracycline only [29]. The same group conducted a prospective-retrospective study using samples from patients with early-stage BC enrolled in the FinHER phase III trial, among which 232 were HER2+. Patients from this subset were randomized to receive trastuzumab, or not, in addition to chemotherapy. Independent pathologists quantified stromal TILs at diagnosis and found that each 10% increase in TILs was significantly associated with decreased distant recurrence rates in patients from the trastuzumab arm [30]. Perez et al. evaluated the association of stromal TILs with recurrence-free survival (RFS) in HER2+ BC patients treated with chemotherapy alone or chemotherapy plus trastuzumab in the N9831 trial. The authors found that stromal TILs, quantified in deciles as above, were prognostically associated with RFS in patients treated with chemotherapy alone but not in patients treated with chemotherapy plus trastuzumab. In contrast to the previous study, high stromal TILs were associated with a lack of benefit from trastuzumab therapy [31]. In the NeoALTTO (Neoadjuvant Lapatinib and/or Trastuzumab Treatment Optimization) trial in which 455 women with HER2+ early-stage BC were randomized in three neoadjuvant treatment arms (trastuzumab, lapatinib, or the combination), followed by adjuvant chemotherapy (paclitaxel and then three cycles of fluorouracil, epirubicin, and cyclophosphamide), Salgado and colleagues evaluated the level of TILs and found that their presence is an independent, positive prognostic marker for both pathological complete response (pCR) and event-free survival (EFS) [28].

Although the majority of TILs are represented by CD8+ cells, other T cell subsets may be present in the TME of BCs, such as Foxp3+ T_regs_. In contrast to CD8 TILs, the pieces of evidence for T_reg_ prognostic/predictive role in BC are few and not always consistent. Liu et al. evaluated the presence of T_regs_ and cytotoxic T lymphocytes (CTLs) by IHC in 1270 cases of invasive BC to assess their association with patient survival, histopathologic features, and molecular subtypes, and found that high infiltration of Foxp3+ T_regs_ in the tumor bed was associated with HER2 overexpression, decreased overall survival (OS), and progression-free survival (PFS) [32]. Similarly, Demir et al. investigated the prognostic and predictive value of Foxp3+ T_regs_ in locally advanced BC patients who received neoadjuvant chemotherapy (NAC) [33]. Again, these investigators reported a higher T_reg_ infiltration in HR-negative/HER2+ tumors. Although this group of patients showed higher pCR rates, they had significantly shorter OS than patients with low T_reg_ infiltration [33]. Contrastingly, Gobert and colleagues reported that the T_reg_ localized within lymphoid infiltrates surrounding the tumor, but not in the tumor bed, was predictive of disease relapse and death. Additionally, in agreement with the above-mentioned studies, they showed that T_reg_ infiltration was significantly correlated with HER2 amplification, and lack of ER/PgR expression [34]. A more recent study, based on the FinHER trial, investigated the clinical significance of total CD4 T cells, T_regs_, and CXCL13 chemokine by using real-time PCR. While high CXCL13 was associated with favorable distant disease-free survival (DDFS), especially in the TNBC, no significant association was detected for CD4 T cells nor Foxp3+ T_regs_ [35].

Using an in-silico approach, Fehrmann’s group used the CIBERSORT bioinformatic tool to estimate, in more than 7000 non-metastatic BC samples, the fraction of 22 immune cell types and assessed their association with pCR, DFS, and OS [36]. They reported an association for an increased fraction of Foxp3+ T_reg_ cells with lower pCR rates and shorter DFS and OS in HER2+ BC cases. One of the possible reasons for a higher T_reg_ infiltration in HER2+ BC may reside in the chemokine and cytokine milieu of the TME of this specific subset. For example, HER2-enriched BCs show high tumor expression of TGF-β, a cytokine also involved in T_reg_ differentiation from CD4+Foxp3- T cells, and of C-C motif chemokine 22 (CCL22), which is involved in T_reg_ recruitment [37]. Interestingly, tumor secretion of CCL22 was found to be positively correlated with intratumor T_reg_ infiltration and with aggressive tumor features such as high histological grade, lack of ER and PgR expression, and HER2 amplification. Univariate analysis showed that CCL22 is an independent prognostic factor for OS and PFS of BC patients [37].

### 3.2. Innate Immune Cells

Macrophages are one of the immune cell subsets more abundantly represented in the TME of most solid tumors, including BC. Tumor-associated macrophages (TAMs) are a heterogeneous population of very plastic cells able to adapt to specific TME and modify their polarization status accordingly [38]. Macrophages infiltrating the tumor are generally considered to be endowed with the so-called M2 pro-tumoral phenotype, although the dichotomic definition of M1 versus M2 macrophages is inaccurate and TAMs often express markers characteristic of both activation states. Nonetheless, their association with tumor aggressiveness and poor prognosis have been well established for several types of cancers [39].

The evidence that TAMs could affect the clinical outcome of BC patients, dates back more than 20 years. Harris’ group, for example, observed a strong relationship between increased macrophage counts and reduced RFS and OS as an independent prognostic variable in a consecutive series of 101 invasive BC [40]. Since then, several groups investigated the role and activities of TAM in BC (reviewed in [18,41,42]), and the prognostic impact of tumor-infiltrating macrophages in BC has been described in several studies. Still, the clinical relevance of TAM infiltration according to the different BC subtypes and the histological localization (stromal and intratumor) is not clear.

To complicate the issue, several markers and different antibodies have been used to assess TAM infiltration over the years, from the initial studies mainly using antibodies directed to the CD68 antigen, to more recent works relying on specific markers more suitable to identify the different TAM populations. Indeed, the CD68 antigen is shared among all monocytes/macrophages but it is not specific for pro-tumoral subpopulations that can be more precisely identified, for example, using antibodies directed to CD163 and CD206 antigens [43]. Macrophage infiltration in BC was also associated with the expression of VEGF and high VEGF expression in TAM is found more frequently in high-grade HR-negative tumors. Additionally, increased levels of VEGF seem to predict poor response to therapy in advanced BC [44,45].

Overall, high TAM infiltration was found to be associated with poor DFS, high histological grade, HR negative status, and advanced disease stages. However, very few studies assessed the prognostic/predictive role of TAM specifically in the HER2+ subset of BC. In recent work, in a small cohort of HER2+ BC patients, Honkanen and colleagues investigated the expression of markers associated with the presence of different macrophage subsets by IHC [46]. They found that a high number of nitric oxide synthase 2 (INOS+) M1-like macrophages, both inside the tumor and at the invasive margins, was significantly associated with better OS, whereas the presence of CD163+ M2-like macrophages, inside the tumor, showed a trend toward poor prognosis [46].

Neutrophils represent another innate immune cell subset often found in the TME, where, under the influence of growth factors and cytokines released by tumor cells, tumor-associated neutrophils (TANs) may, like macrophages, undergo polarization, and become either pro-tumorigenic (N2 phenotype) or pro-inflammatory/antitumorigenic (N1 phenotype) [47]. However, their role and prognostic/predictive value in human cancers, including BC, have not been systematically investigated. In recent work, Soto-Perez-de-Celis and colleagues analyzed patients with stage I-III BC, including HR+/HER2-, HER2+, and TNBC patients. TAN+ infiltration, scored as >1 TAN per 10 HPF, was detected in 88% of TNBC, 53% of HER2+, and 5% in HR+/HER2- tumors. HER2 expression and tumor grade were associated with TAN positivity, whereas in a multivariate analysis, HR negativity was the only predictor of TAN positivity [48]. More consolidated is the evidence that a high neutrophil to lymphocyte ratio (NLR) in the peripheral blood of BC patients is associated with poor outcome [49,50] and may have predictive value, being correlated with high recurrence and poor survival after recurrence [51,52], but no specific studies have been published for HER2+ BC.

It should be noted that neutrophils share several markers with the granulocytic subset of the so-called myeloid-derived suppressor cells (MDSCs), a heterogeneous immature population of myeloid cells characterized by suppressive activities on immune cells such as T cells, dendritic cells, and NK cells. In humans, MDSCs are positive for the markers CD33 and CD11b and express low/negative levels of HLA-DR [53]. The role of MDSCs in BC has been widely investigated and confirmed in several preclinical mouse models [54], whereas evidence for a role in human BC is mainly provided by studies assessing MDSCs in peripheral blood. However, a few works also investigated tumor-infiltrating MDSCs. Hix and colleagues, studying the role of STAT1 in BC, demonstrated that the expression of this transcription factor and the recruitment of CD33+ myeloid cells correlate with increased disease progression from ductal carcinoma in situ to invasive carcinoma [55]. More recently, Sangaletti et al. showed that ECM3 BCs, which are high-grade tumors with EMT features, i.e., reduced treatment response and poor prognosis [56], are characterized by an enrichment of infiltrating CD33+ myeloid cells in direct contact with cancer cells [57].

The studies on circulating MDSCs indicate that the percentage of these cells in patients’ blood correlates with disease burden and may therefore represent a promising prognostic biomarker. In a study on different types of solid tumors, including BC, Diaz-Montero et al. showed a significant correlation between circulating MDSCs and clinical tumor stage, and in particular, in patients with stage IV BCs they were found to directly correlate with metastatic tumor burden [58]. However, no specific studies have been reported on MDSC significance in Her2+ BC.

## 4. Recent Advance in Analyzing (Breast Cancer) Landscape

The dynamic and spatial nature of tumor-stroma interactions, as well as the existence of possible biases such as those related to the sampling of biopsies (the biopsy may not be reliable for the entire tumor mass), suggested adopting more complex omic technologies to dynamically capture both the immune microenvironment composition and the clonal evolution of tumor cells. This analysis, done at the time of diagnosis, at relapse, or metastatic disease, could inform on clonal evolution, but it can also give information on how the TME is similar or distant from that of the primary tumor. In the latter case, it could suggest that distant metastases could be the result of tumor adaptation to new local environmental cues offered by the target organ. The latter might enable key pathways for survival, proliferation, and DNA repair. For example, the oxygen level is different among different tissues and the different hypoxic conditions may have a direct impact on the expression of relevant programs such as Myc and DNA repair genes, as we have recently shown in the context of hematologic tumors [59].

Single-cell RNA sequencing (scRNA-seq) technologies represent a potent tool to study inter-and intra-tumor heterogeneity in both tumor cells and the stromal/immune compartment. Concerning the cellular origin of the different BC subtypes, scRNAseq analysis has first revealed that this heterogeneity might rely on the heterogeneity of normal breast epithelial cells, hence their transformation might generate the different BC subtypes [60,61]. On this line, Chung et al. performed a scRNAseq and a bulk RNAseq analysis on 11 patients representing four subtypes of breast cancers: luminal A, luminal B, HER2, and TNBC [62]. They identified heterogeneity and core gene expression signatures for subtype-specific BC cells, as well as a distinct immune system status in each tumor sample. HER2 and TNBC were enriched in stemness and recurrence signature as well as in T lymphocytes with the expression of regulatory T cell markers.

How stromal/immune cells interact with BC cells creating micro-niches sustaining tumor growth, escape, or survival can be potentially identified through Spatial Transcriptomics technology [63]. Here, the definition of specific regions of interest (ROI) using antibodies to relevant populations (i.e., cytokeratins, CD markers, a-SMA, PDGFRβ, or others) would allow testing how the stromal/immune microenvironment could generate areas of transcriptional heterogeneity, acting on key genetic and metabolic pathways supporting tumor growth and treatment resistance. An interesting application of the DSP approach was the testing of immune changes induced by trastuzumab after a single dose of the agent [64], a part which will be discussed in the section below.

## 5. Changes in the Immune Microenvironment of HER2+ Breast Cancer upon Treatment

Among the different intrinsic BC subtypes, HER2-enriched tumors are those characterized by a higher pCR rate upon treatment. This might rely on the immunogenicity of HER2, and certainly, the response to trastuzumab depends on the activation of immune cells; additionally, the frequency of TILs at baseline is associated with pCR and EFS [65]. However, HER2+ BC is a highly heterogeneous disease in which the differential expression of HR, along with the different infiltrating immune cell subsets, predicts the response to trastuzumab-based treatment (Figure 1). One of the reasons for HR+HER2+ patients showing a reduced pCR rate relies on the fact that during sustained HER2 inhibition, ER functions as a key escape/survival pathway [66,67]. Additionally, it has been shown that hormones exert negative effects on immune cell activation by reducing the transcription of SerpinB9/proteinase inhibitor 9, a granzyme B inhibitor known to decrease susceptibility of HR+ BC cells to NK and CD8+ T-cell cytotoxicity in vitro (reviewed in Griguolo et al. [68]). A complete blockade of the HER2 network, coupled with ER inhibition, may therefore provide optimal therapy in selected HR+HER2+ patients.

As the pCR is a strong surrogate of PFS, the neoadjuvant setting in HER2+BC represents a favorable condition to develop new biomarkers of treatment response or for dose de-escalation. Specifically, some studies have been designed to characterize immune and microenvironmental changes occurring after a single cycle of HER2-targeted therapy. Evaluation of such changes has been performed using different technologies: multiplex spatial IHC [69], total GEP/RNAseq analysis with deconvolution of immune cells (i.e., using the CIBERSORT tool) [70,71,72], or DSP [64]. The multicenter study (03-311 and 211B trials) from Varadan et al. was the first showing the immunomodulatory effect of brief treatment with trastuzumab. Their Gene Expression Profile (GEP) analysis, performed on biopsies obtained at baseline and day 14 after trastuzumab, revealed an immune signature that, at day 14 and not at baseline, was predictive of response in HER2-enriched tumors but not in HER2-luminal or basal subtypes [70]. CD4+ follicular helper T cells and PD-1 expression were mainly responsible for the immunological changes observed. In the NeoALLTO trial already mentioned above, RNAseq analysis of tumor biopsies at baseline and day 14 allowed to the classification of patients as immune-poor and immune-enriched, with the last being associated with pCR and improved EFS [72]. Immune-enriched pre-treatment samples showed a high infiltration by dendritic cells (DCs), NKs, and CD8+ T cells, including tissue-resident memory cells, a finding that was suggestive of a requirement for key immune cell subsets to elicit anti-HER2 associated responses. More recently, Pizzamiglio et al. explored the clinical value of 5 immune metagenes and 22 tumor-infiltrating immune cells in HER2+ BC patient subgroups based on bulk GEP analysis from the NeoALTTO trial (Table 1) in relation to pCR, EFS, clinical characteristics, and molecular classifiers [73]. The increased fraction of γδ T cells was an independent predictive factor of pCR in the overall study cohort. Furthermore, a high expression of the STAT1 metagene predicted a better EFS in the overall study cohort. Considering HER2-addicted and non-addicted tumors, it was found that increased expression of STAT1 and hematopoietic cell kinase (HCK), paralleled by an increased fraction of γδ T cells, define the HER2-addicted tumors with the highest chance of treatment response [73].

The study by Griguolo et al. on the PAMELA trial (Table 1) was based on multiplex IHC and showed a significant modification in the immune infiltrate at day 15 post-HER2-treatment, that was characterized by an increased overall density of CD3+, CD4+, CD8+, Ki67+, and Foxp3+ cells in the intra-tumor and proximal tumor areas [69]. They also confirmed a strong TIL infiltration at day 15 in HR- HER2+ tumors (12.1% compared to baseline), whereas in HR+ tumors, the difference between baseline and day 15 was only 3.2%. As in the paper from Varadan, this study reported an association between pCR and the immune infiltrate at day 15, however, in this case, the strongest impact on pCR was achieved when immune cells spatially interacted with tumor cells. The authors also reported a decreased TILs infiltration at surgery in tumors achieving pCR, but not in patients with residual disease, suggesting a possible beneficial effect from the use of immune checkpoint inhibitors for the previous. Interestingly, the GEP analysis of TILs showed enrichment in genes associated with T cell activation, among them, MS4A1, PD1, CD8A, CD19, IKBKE, IDO1, TAP1, TYMP, CD3G, and LAG3. The expression of markers such as IDO1 might be suggestive of a progressive establishment of immune suppression, which might be relevant for patients characterized by residual disease [69].

The relevance concerning the immune cell spatial distribution other than the sole frequency has been investigated in-depth in a recent paper published by McNamara et al. [64]. In this work, the authors analyzed core biopsies obtained from 57 patients {subdivided between a training and a validation set, from the TRIO-US B07 clinical trial (Table 1)} through DSP. They selected multiple ROIs per tissue sample based on pan-CK/CD45 and double-strand DNA immunofluorescence. The markers that were most associated with pCR at the on-treatment time point were immune-related, specifically CD45 and CD8. Consistent with the efficacy of CD45 to predict pCR, the authors next sought to determine whether the CD45 measurement from DSP correlated with CD45 IHC and stromal TILs. Both markers proved to be associated with pCR, and in the case of CD45, they identified a threshold of 20% positive cells.

Transcriptional profiles of pre-and post-treatment tumor samples from 17 HER2+ BC patients were analyzed on the Illumina platform by Triulzi et al. (Table 1) [71]. They compared the GEP of pretreatment tumors and biopsies obtained after one cycle of trastuzumab (day 21). Responders were defined according to the reduction in tumor dimensions, at least 20%, between post- and pre-treatment (clinical response), and to the reduction of Ki67 staining. In this case, patients were considered to be responders if the number of Ki67-positive cells in the post-treatment biopsy decreased by at least 50% compared with the pre-treatment sample. The GEP revealed that tumors from clinically responder patients, who also showed a decrease in Ki67, were characterized by the enrichment of an immune-related signature with MHC-II, interferon (IFN)-related genes, and T cell markers, as leading genes. Deconvolution analysis of immune cells through CIBERSORT indicated that the increase in MHC-II gene expression was dependent on the enrichment in M1 macrophages. To confirm that the change in MHC-II expression was due to the recruitment of immune cells in the TME by the effects of trastuzumab, they moved to mouse models and showed that, among the immune cells recruited upon trastuzumab treatment, half of them were macrophages [71].

Overall, these studies shed some light on the immune markers associated with trastuzumab response, confirming that immune cell enrichment is generally associated with pCR and suggesting that the longitudinal analysis of pre-treatment and on-treatment biopsies could be a reliable approach for the discovery of new biomarkers. A more comprehensive list of clinical trials that have investigated changes in the tumor immune landscape upon treatment in HER2+ BC, including the above-mentioned studies and others, is shown in Table 1 below.

## 6. Targeting the Tumor Microenvironment in HER2+ Breast Cancer

One of the major issues in the clinical management of HER2 tumors is the development of therapy resistance that could be due to different factors, most of which are directly related to escape molecular mechanisms exerted by the cancer cells [74,75]. One of the mechanisms associated with trastuzumab resistance involves cyclin D1-CDK4-mediated proliferation [76,77]. The combination of CDK4/6 inhibitors plus trastuzumab is currently under clinical investigation. Interestingly, in a pre-clinical translational study based on the use of MMTV-Neu mammary tumor model and single-cell profiling, Wang et al. showed the enrichment of an immunosuppressive immature myeloid cell population infiltrating trastuzumab+CDK4/6 inhibitors-resistant tumors [78]. Notably, adding cabozantinib, a tyrosine kinase inhibitor that also targets immature myeloid cells, sensitized tumors to immune checkpoint blockades, preventing resistance. This work, as well as other pieces of evidence, indicates that the TME may contribute to trastuzumab resistance, either in the case of intrinsic/initial resistance or acquired resistance. Resistance to anti-HER2 treatments could also be mediated by immune-suppressive mechanisms relying on the acquisition of the PD-L1/PD1 axis.

In a murine mouse model expressing oncogenic rat ErbB-2, Stagg et al. demonstrate a role for both CD8 T cells and NK cells in the response to anti-HER2 therapy and show that anti-PD1 or anti-4-1BB antibodies are able to increase its therapeutic activity in immunocompetent mice [79]. Moving to clinical studies, different trials have been done and are ongoing to evaluate the activity of anti-PD-1 and anti-PD-L1 inhibitors in HER2+ BC patients (reviewed in [68]). In a single-arm study, combination therapy with pembrolizumab plus trastuzumab was well tolerated in advanced HER2+ BC patients. Durable anti-tumor activity was especially demonstrated in patients characterized by PD-L1 positivity and higher T cell infiltration [80]. However, the absence of a control group, as well as the small size of patients enrolled in this study (n = 58), suggests that larger randomized trials will be required to definitively prove the efficacy of immune-checkpoint blockade in combination with trastuzumab [80].

Furthermore, the data obtained so far indicate that the evaluation of PDL-1 expression in HER2+ BC biopsies could be useful to select the subjects that can realistically benefit from the treatments [81]. Indeed, Emens et al. found targeting PDL-1 with atezolizumab in combination with trastuzumab emtansine (T-DM1) did not demonstrate a clinically significant PFS in HER2+ advanced BC patients, and that this combo therapy was correlated with detrimental effects. On the other hand, higher PFS and overall response rate (ORR) were observed in patients with HER2+/PD-L1+ BCs. Further investigations are required, as the the positive effect observed in a small fraction of patients is uncertain and probably dependent on a pre-existing immunity [82].

After standard therapy, targeting other immune biomarkers in the primary tumor, such as CTLA-4 that is often detected in inflamed breast cancers, TIM3 and LAG3 that are expressed in early-stage HER2+ BC [83,84], or IDO and CD73 that are identified in TILs after treatment with anti-PDL-1 antibodies [85,86], could revert resistance to standard treatments and improve clinical response and OS [87]. In this context, preclinical studies showed that anti-HER2 blockade activates a strong antitumor immune response and renders HER2+ tumors highly sensitive to dual CTLA-4/PD-1 checkpoint inhibition [84]. Furthermore, the combination of T-DM1 with CTLA-4/PD-1 blocking antibodies enhances T cell infiltration and promotes tumor rejection and immunological memory formation [84].

**Table 1:** Clinical studies of HER2+ BC and modulation of immune TME upon treatment.

Study	Strategy	Therapy	Evaluation of Treatment	Immunological Changes	Clinical Response	Ref
FinHER	H&E	Docetaxel or vinorelbine + FEC + trastuzumab	pre and post-treatment	TILs	DDFS	[30]
GeparSixto	H&E and mRNA analysis	Paclitaxel and non pegylated liposomal doxorubicin with trastuzumab and lapatinib	pre and post-treatment	TILs and lymphocyte-predominant BC	pCR	[64]
03-311 and 211B	GEP analysis	Trastuzumab	pre-treatment and 14 days after treatment	CD4+ Follicular helper T-cells and PD-1	pCR	[69]
PAMELA and LPT109096	H&E	PAMELA: NAT with lapatinib and trastuzumab (±hormonal therapy). LPT109096: lapatinib, trastuzumabor both and chemotherapy	pre-treatment and 15 days after treatment	Cellularity and TILs (CelTIL)	pCR	[70]
TRUP WOO	GEP analysis	Trastuzumab and paclitaxel	pre-treatment and 21 days after treatment	MHC-II, interferon-related genes, and T cell markers	Ki67 and MHCII	[71]
NeoALLTO and PAMELA	RNAseq analysis	NeoALLTO: NAT with trastuzumab, lapatinib, or combination with paclitaxel, followed by FEC after surgery. PAMELA: NAT with lapatinib and trastuzumab (±hormonal therapy).	pre-treatment and 14 days after treatment	DCs, NKs, CD8+, and resident memory cells	pCR and EFS	[72]
PAMELA	Multiplex spatial IHC	Lapatinib-trastuzumab	pre-treatment and 15 days after treatment	CD3+, CD4+, CD8+, Ki67+, and Foxp3+	pCR and immune infiltrate	[81]
TRIO-US B07	Digital spatial profiling	Lapatinib-trastuzumab	pre-treatment and 14–21 days after treatment	CD45 and CD8	pCR	[87]

Footnotes: H&E: hematoxylin and eosin; GEP: gene expression profile; IHC: immunohistochemistry; FEC: 5-fluorouracil epirubicin cyclophosphamide; NAT: neoadjuvant therapy; TILs: tumor-infiltrating lymphocytes; Bc: breast cancer; DCs: dendritic cells; NKs natural killer cells; DDFS: distant disease-free survival; pCR: pathological complete response; PFS: progression-free survival; EFS event-free survival.

It is well established that the antitumor activity of anti-HER2 antibodies is also associated with the Fcγ receptor (FcγR) expressed on macrophages, NK, and DC cells. The binding of trastuzumab with FcγR establishes the efficacy of therapy activating the phagocytic activity of macrophages [88,89]. Accordingly, expansion of iNOS+ M1-macrophages and CD8+ T cells in the TME is a positive indicator of prognosis and efficacy of anti-HER2 treatments [46]. However, it has been shown that neoadjuvant chemotherapy induces the expansion of TAMs with an immune-suppressive M2 phenotype and reduces the amount of Ki67+ T cells, leading to immune tolerance.

In this context, Su et al. demonstrated that after antibody-dependent cellular phagocytosis (ADCP), macrophages inhibit the antibody-dependent cellular cytotoxicity (ADCC) activity of NK cells and T cell-mediated cytotoxicity through their up-regulation of both PD-L1 and IDO. The authors elegantly reported that the combined treatment with the anti-HER2 antibody and inhibitors of PD-L1 and IDO enhances anti-tumor immunity and anti-HER2 therapeutic efficacy in a mouse model [90]. The up-regulation of PDL-1 and IDO1 was also confirmed in TAMs of HER2+ BC patients after trastuzumab neoadjuvant therapy, and their expression correlated with poor therapeutic response [90].

Interestingly, another family of drugs potentially able to modulate the different types of myeloid cells is that of bisphosphonates. Several clinical trials showed that neoadjuvant therapy based on Zoledronic Acid (ZA), a third generation of bisphosphonate, increases the OS of BC patients [91]. Crocamo et al. evaluated the efficacy of ZA as neoadjuvant therapy in HER2+ BC patients with a high tumor burden. The therapeutic efficacy of ZA, in combination with standard chemotherapy plus anti-HER2 targeted therapy, was well tolerated in vivo, and the reported results suggest that such a combinatorial treatment strategy could overcome resistance to standard anti-HER2 agents in HR+/HER2+ BCs [92].

In addition, Christmas et al. showed that targeting MDSC migration using HDAC inhibitor in combination with anti-PD-1, anti-CTLA-4, or both, significantly reduces MDSC infiltration and tumor growth in HER2/neu transgenic mice [93].

## 7. Conclusions

Intra-and inter-tumor heterogeneity characterizes BC disease at different levels, both in intrinsic cancer cell features and in the surrounding TME that can be populated by different stromal and immune cell types. As TNBC and HER2+ tumors are the most immune infiltrated, the role of immune cells in their clinical outcome and response to therapy is expected. The neoadjuvant setting, largely applied to HER2+BCs, represents a favorable condition to discover new immune/microenvironmental biomarkers of treatment response or dose de-escalation. Indeed, several trials have been designed to characterize the immune and microenvironmental changes occurring after a single cycle of HER2-targeted therapy, and the development of new technologies allowing an in-depth dissection of the local TME is expected to further improve the development of such immune biomarkers.

## Data Availability

Not applicable.
